# Circulating IL-10 is compromised in patients predisposed to developing and in patients with severe knee osteoarthritis

**DOI:** 10.1038/s41598-021-81382-6

**Published:** 2021-01-19

**Authors:** Tyler Barker, Victoria E. Rogers, Vanessa T. Henriksen, Roy H. Trawick, Nathan G. Momberger, G. Lynn Rasmussen

**Affiliations:** 1 Precision Genomics, Intermountain Healthcare, 383 W. Vine Street, Suite #300, Murray, UT 84107 USA; 2 Nutrition and Integrative Physiology, University of Utah, Salt Lake City, UT 84112 USA; 3 The Orthopedic Specialty Hospital, Murray, UT 84107 USA; 4 The Orthopedic Specialty Clinic, Murray, UT 84107 USA

**Keywords:** Physiology, Health care

## Abstract

The purpose of this investigation was to identify if serum interleukin (IL)-10 and tumor necrosis factor (TNF)-α concentrations and their ratio (IL-10/TNF-α) are altered in subjects predisposed to developing knee osteoarthritis following ligamentous injury and in those with severe knee osteoarthritis. Serum IL-10 and TNF-α concentrations were measured in four groups of subjects (*n* = 218): (1) reportedly-healthy and non-injured control subjects (CON; *n* = 92), (2) subjects scheduled to undergo anterior cruciate ligament surgery (ACL; *n* = 42), (3) non-surgical subjects with knee osteoarthritis (OA; *n* = 60), and (4) subjects with knee osteoarthritis scheduled to undergo total knee arthroplasty (TKA; *n* = 24). X-ray images were used to grade the severity of knee osteoarthritis. Serum IL-10 and the serum IL-10/TNF-α ratio were significantly lower while serum TNF-α was not significantly perturbed with severe compared to moderate knee osteoarthritis (i.e., Kellgren-Lawrence grade 4 vs. 3, respectively). Serum IL-10 was significantly lower in the absence of serum TNF-α alterations in the ACL group. We conclude that serum IL-10 concentrations are compromised in subjects predisposed to developing knee osteoarthritis following ligamentous trauma and in subjects with radiographic evidence of severe knee osteoarthritis.

## Introduction

Cytokines are pleiotropic and multifunctional signaling molecules that regulate innate and adaptive immunities, inflammation, and hematopoiesis. Of the various cytokines, tumor necrosis factor (TNF)-α is a prototypical pro-inflammatory cytokine and interleukin (IL)-10 is a quintessential anti-inflammatory cytokine that reciprocally down-regulates pro-inflammatory cytokine production. Following one of the most commonly injured ligaments in the knee, the anterior cruciate ligament, reports indicate a local (i.e., synovial fluid) increase in a variety of cytokines^1–4^, including IL-10 and TNF-α^5–10^. Although results are inconsistent^11^, IL-10 and/or TNF-α may be influential on the development of osteoarthritis^12,13^.

The local increase in IL-10 appears to be transient^5^ while the increase in TNF-α may persist years after an anterior cruciate ligament injury^12^, thereby suggesting a temporal shift in the anti- to pro-inflammatory cytokine balance in favor of the latter. The TNF-α increase after an anterior cruciate ligament injury associates with the proteolysis of aggrecan and type II collagen^12^, and thus, could contribute to trauma-induced joint degradation as found in a porcine model^14^. With the exception of data regarding serum IL-6^3^, studies identifying if IL-10 and TNF-α concentrations or their concentration balance are perturbed in the circulation following an anterior cruciate ligament injury compared to non-injured control subjects and to those with degenerative knee joint disease are surprisingly sparse^12,15^ or lacking, respectively.

Knee osteoarthritis is a degenerative joint condition, a leading cause of disability in the United States^16^, and is characterized by low-grade inflammation^17,18^. The localized presence of TNF-α could increase the risk of osteophyte progression, joint space narrowing^19^, cartilage degradation^20^, and lower IL-10 expression^21^. IL-10 decreases TNF-α production and TNF-α mediated events associated with osteoarthritis development^22–25^, thereby suggesting the pathophysiological importance of a regulated anti- and pro-inflammatory cytokine balance on joint integrity. Although an increased production of IL-10 and TNF-α from whole blood associates with the progression in knee osteoarthritis^26^, and preliminary results indicate that local and circulating cytokine concentrations decrease with severe compared to moderate knee osteoarthritis^27,28^, it is unknown if the circulating pro- to anti-inflammatory cytokine balance (i.e., ratio) is altered with the radiographic severity in knee osteoarthritis. In septic patients, the IL-10/TNF-α ratio has been previously suggested to serve as an anti-inflammatory index^29^.

The purpose of this investigation was to identify if serum IL-10 and TNF-α concentrations and their ratio (i.e., IL-10/TNF-α) are altered in subjects predisposed to developing knee osteoarthritis following ligamentous trauma and in those with severe knee osteoarthritis. We hypothesized that serum IL-10 and TNF-α concentrations are higher following an anterior cruciate ligament injury and that the serum IL-10/TNF-α concentration ratio is lower with severe knee osteoarthritis. The anticipated findings could advance our understanding of systemic cytokine alterations following ligamentous trauma in the knee and with severe knee osteoarthritis, both of which, are characterized by inflammatory-driven processes that contribute to localized pathophysiology.

## Methods

Serum cytokine concentrations were measured in four groups of subjects (*n* = 218): (1) reportedly-healthy and non-injured control subjects (CON; *n* = 92; ≥ 18 years), (2) subjects scheduled to undergo primary, unilateral anterior cruciate ligament surgery (ACL; *n* = 42; 18–45 years), (3) non-surgical subjects with unilateral knee osteoarthritis (OA; *n* = 60; 18–60 years), and (4) subjects with end-stage knee osteoarthritis scheduled to undergo a primary, unilateral total knee arthroplasty (TKA; *n* = 24; ≥ 18 years). Baseline (i.e., prior to intervention in previous studies, where applicable) data from some of the CON^30,31^, ACL^9,32^, and OA^33,34^ subjects have been previously published. All subjects were informed of and provided written and verbal consent to the study protocols and procedures. This study was approved by the Institutional Review Board at Intermountain Healthcare (Salt Lake City, UT USA). All experiments were performed in accordance with relevant guidelines and regulations.

All subjects were recruited from the physician clinics and other clinical departments at The Orthopedic Specialty Hospital (Murray, UT USA). For all study groups, modestly active (i.e., 30 min of continuous physical activity ≥ 3 times per week) subjects were recruited for study participation. To minimize the presence of co-morbidities, potential CON, ACL, OA, and TKA subjects were excluded from participation if they had a history of metabolic bone disease, skeletal muscle pathology, cardiac or peripheral cardiovascular system abnormality, clotting disorder, coronary artery disease, peripheral vascular disease, stroke, cancer, high cholesterol or triglycerides, or high blood pressure. Severely obese [body mass index (BMI) > 40 kg/m^2^], smokers, or subjects that suffered a leg injury during the previous year that required the use of crutches (with the exception to the ligamentous injury sustained in the ACL group subjects) for more than 1-week in the past year were excluded from study participation as well. Potential subjects were also excluded from participation if they were using warfarin or other anti-coagulants, cholesterol lowering medication, corticosteroid medication, orlistat, phenobarbital, phenytoin, or thiazide, or a daily dietary supplement or vitamin during the previous year, diagnosed with diabetes mellitus, impaired liver or kidney function, or pregnant. Also, no subjects were currently receiving any physician-prescribed or -guided medications based on an underlying condition upon study enrollment, including intra-articular injections.

In addition to those above, other condition specific inclusion and exclusion criteria were implemented. In the OA group, subjects were excluded from participation if they had a recent (within 2 years) surgery on the involved or non-involved leg, planning on undergoing surgery to treat knee osteoarthritis, or received any osteoarthritis specific medication or treatment prior to enrollment. To be included in the OA groups, subjects also had to possess: (1) a Western Ontario and McMaster Universities Osteoarthritis Index pain score ≥ 2 on all of the five questions in its subsection, (2) evidence of muscular weakness (i.e., deficit in peak isokinetic concentric knee extension or flexion torque at 60°/s) in the involved leg compared to the non-involved leg, and (3) a Kellgren–Lawrence (KL) grade ≥ 2 was scored in the involved knee. Therefore, the OA group consisted of subjects with signs and symptoms of unilateral knee osteoarthritis (i.e., pain, muscular weakness, and radiographic evidence of mild-to-severe knee osteoarthritis in the involved leg) that were not scheduled or planning to undergo total knee arthroplasty or any other knee surgery in the foreseeable future. In the TKA group, subjects were excluded from participation if they had a recent (within 2 years) surgery on the involved or non-involved leg, experiencing bilateral symptoms of tibiofemoral osteoarthritis, or planning on undergoing elective surgery on the non-involved leg in the foreseeable future.

### Study protocol

Serum cytokine data from a fasting (10–12 h) blood draw sample obtained at baseline, and if applicable, prior to any surgical procedures (i.e., ACL or TKA) or study interventions (e.g., vitamin D or placebo), were aggregated for this investigation. Subjects were instructed to refrain from exercise and using aspirin, ibuprofen, naproxen sodium, acetaminophen, or other anti-inflammatory agents 72-h prior to providing a fasting blood draw sample. After collection and coagulation, samples were centrifuged at 1100 g for 10 min and aliquoted into several small cryovials. Aliquots were stored at − 80° C until analysis.

### Analytical procedures

#### Serum cytokines

Serum cytokine concentrations were determined using the multiplex technology of Luminex (MAGPix, Austin, TX USA) with high sensitivity (EMD Millipore, Billerica, MA USA; catalog #: HSCYMAG60SPMX13) at The Orthopedic Specialty Hospital (Murray, UT USA). Inter-assay precision is < 15% and intra-assay precision is < 10%. The minimum detectable concentration is 0.56 pg/mL and 0.16 pg/mL for IL-10 and TNF-α, respectively. Based on the objectives and hypotheses of this study, we only examined IL-10 and TNF-α from the high sensitivity panel.

#### Radiographic evidence of knee OA

Weight bearing X-ray images were obtained on each knee in the anterior–posterior view at 45° of knee flexion. Knee joint space was analyzed using ImageJ software (National Institutes of Health). Severity of knee OA was classified according to the KL scoring criteria: 0, no osteophytes or joint-space narrowing; 1, questionable osteophyte indicating possible OA; 2, definite osteophyte, no joint-narrowing (compared to the non-symptomatic knee) indicating mild OA; 3, ≤ 50% joint-space narrowing (compared to the non-symptomatic knee) indicating moderate OA; and 4, > 50% joint-space narrowing (compared to the non-symptomatic knee) indicating severe OA^35^. X-ray images were part of the previous study protocols or as a standard of care procedure in the OA and TKA groups but not in the CON and ACL groups. Therefore, radiographic images were not available for all the subjects in the CON and ACL groups (see Table 1).

**Table 1:** Subject characteristics.

	CON	ACL	OA	TKA	*p* value
*n* (m/f)	92 (63/29)	42 (25/17)	60 (24/36)	24 (9/15)	< 0.01
Age (y)	32 (13)	33 (12)	52 (13)^a,b^	64 (7)^a,b,c^	< 0.01
Height (cm)	174 (14)	173 (15)	168 (14)^a^	163 (15)^a^	< 0.01
Body mass (kg)	78.2 (21.6)	84.4 (22.9)	90.8 (32.4)^a^	93.1 (35.6)^a^	< 0.01
BMI (kg/m^2^)	25.4 (4.4)	28.0 (6.5)	31.6 (8.6)^a,b^	32.2 (10.7)^a,b^	< 0.01
KL grade					< 0.01
0 (*n* = 25)	10 (10.9%)	15 (35.7%)	0	0	
1 (*n* = 18)	3 (3.3%)	15 (35.7%)	0	0	
2 (*n* = 8)	–	3 (7.1%)	5 (8.3%)	0	
3 (*n* = 49)	–	–	41 (68.3%)	8 (33.3%)	
4 (*n* = 30)	–	–	14 (23.3%)	16 (66.7%)	
n (%)	13 (14.1%)	33 (78.6%)	60 (100%)	24 (100%)	
Days from injury (d)	NA	17.5 (37.0)	NA	NA	

Data presented as median (interquartile range). ^a^*p* < 0.05 versus CON; ^b^*p* < 0.05 versus ACL; ^c^*p* < 0.05 versus OA. *NA* not applicable; – not available.

### Statistical analysis

Data were checked for normality with a Shapiro–Wilk test prior to statistical analysis. When necessary, rank transformations were performed on data to achieve normality before analysis. Statistical significance of data between groups were assessed with an analysis of variance (ANOVA) and followed by a Tukey test on multiple pairwise comparisons when appropriate. Group associations with categorical variables were assessed with separate Pearson Chi-Square tests. A Pearson Product moment linear correlation was performed to examine the association between variables. Age, gender, and BMI were used as covariates. Significance was set at *p* < 0.05. All statistical analyses were performed with SYSTAT (version 13.1, Chicago, IL USA).

### Ethics approval and consent to participate

This study was approved by the Institutional Review Board at Intermountain Healthcare (Salt Lake City, UT USA).

## Results

### Subject characteristics

Subject characteristics were not significantly different between the CON and ACL groups, and except for age (*p* < 0.05), not significantly different between the OA and TKA groups (Table 1). Age, body mass, and BMI (all *p* < 0.05) were higher and height (*p* < 0.05) was lower in the OA and TKA compared to the CON and/or ACL groups. Radiographic images indicate that the severity in knee osteoarthritis was greater (*p* < 0.05) in the TKA compared to the CON, ACL, and OA groups. Of note, ~ 68% in the OA and ~ 33% in the TKA group possessed moderate knee osteoarthritis (i.e., KL grade of 3), while conversely, ~ 23% in the OA and ~ 67% in the TKA group displayed severe knee osteoarthritis (i.e., KL grade of 4; see Table 1).

### Serum cytokine concentrations as a function of concomitant injuries with an anterior cruciate ligament rupture

Additional trauma or damage to the surrounding knee tissue concurrently with a ruptured anterior cruciate ligament increases localized cytokine concentrations^8^. In the present study, however, serum IL-10 and TNF-α concentrations were not significantly different with compared to without concomitant damage to the localized surrounding tissue found and repaired during arthroscopic surgery in the ACL group (Table 2), a finding consistent with a prior investigation performed in synovial fluid^36^. Therefore, we subsequently combined those with and without additional knee joint trauma in the ACL group. Additionally, some circulating and localized cytokine concentrations are dependent on the time from anterior cruciate ligament injury occurrence^5,6,9^. Contrasting with previous data^5,6,9^, serum IL-10 (*r* =  − 0.01, *p* = 0.94, *data not shown*) and TNF-α (*r* = 0.09, *p* = 0.57, *data not shown*) concentrations were not correlated with the days from anterior cruciate ligament injury occurrence (range 1–348 days).

**Table 2:** Serum IL-10 and TNF-α concentrations with and without additional surgical procedures performed during ACL reconstructive surgery.

	Without	With	*p* value
*n*	22	20	0.76
Additional surgical procedures (*n*)	0	20	
Lateral meniscus repair (*n*)		3	
Medial meniscus repair (*n*)		6	
Partial lateral meniscectomy (*n*)		4	
Partial medial meniscectomy (*n*)		6	
Posterior oblique ligament repair (*n*)		1	
Serum IL-10 (pg/mL)	6.14 (9.49)	7.80 (25.3)	0.19
Serum TNF-α (pg/mL)	4.80 (5.14)	4.76 (9.21)	0.64

Data presented as median (interquartile range).

### Serum cytokine concentrations as a function of group (CON, ACL, OA, and TKA)

**Figure 1:**
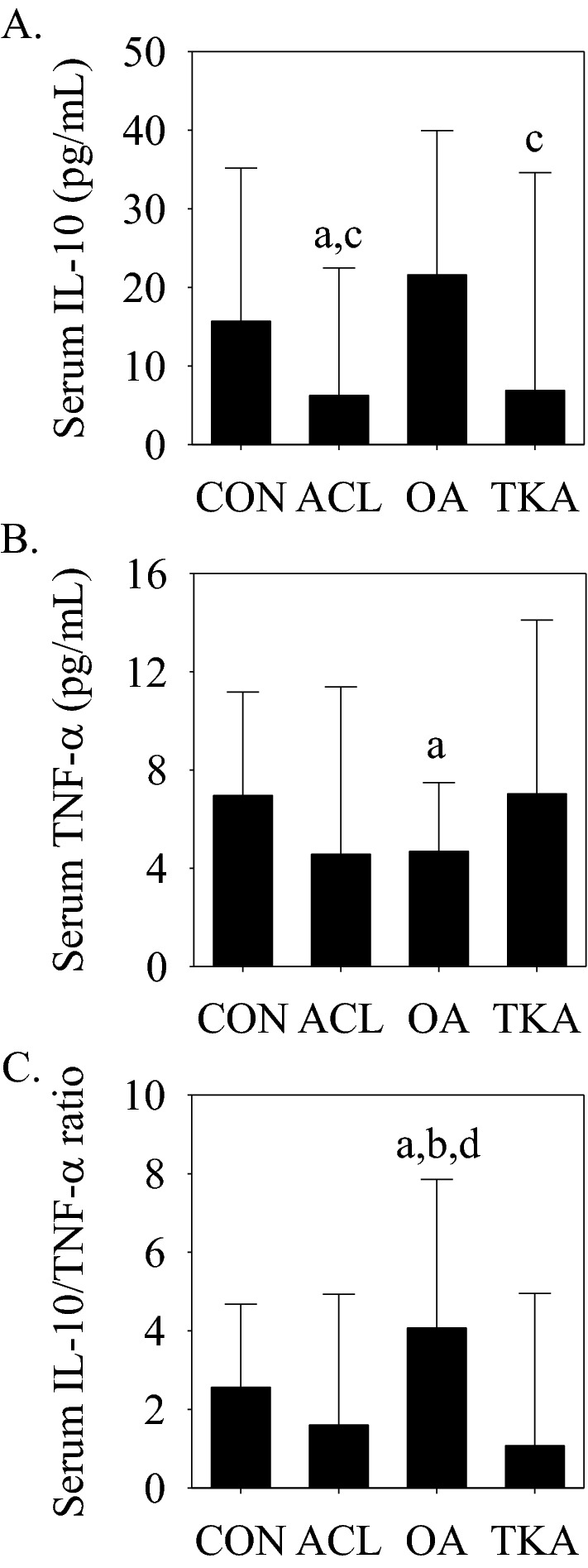
Serum IL-10 and TNF-α concentrations and the serum IL-10/TNF-α ratio in the CON, ACL, OA, and TKA groups. (**A**) Serum IL-10 concentrations were significantly lower in the ACL (^a^*p* < 0.05 vs. CON and ^c^*p* < 0.05 vs. OA) and TKA (^c^*p* < 0.05 vs. OA) groups. (**B**) Serum TNF-α concentrations were significantly (^a^*p* < 0.05 vs. CON) lower in the OA group. (**C**) The serum IL-10/TNF-α ratio was significantly (^a^*p* < 0.05 vs. CON, ^b^*p* < 0.05 vs. ACL, and ^d^*p* < 0.05 vs. TKA) higher in the OA group. Data presented as median (interquartile range).

A primary outcome of this investigation was the difference in serum IL-10 and TNF-α concentrations and the IL-10/TNF-α ratio between the ACL, CON, and OA groups. However, since the investigation also included subjects undergoing knee arthroplasty, we included a TKA group in the comparisons. Serum IL-10 concentrations were not significantly different between the CON and OA groups (Fig. 1A), but concentrations were lower in the ACL (*p* < 0.05 vs. CON and OA) and TKA (*p* < 0.05 vs. OA) groups. Serum TNF-α concentrations were lower (*p* < 0.05) in the OA compared to the CON group (Fig. 1B). The serum IL-10/TNF-α ratio was significantly higher in the OA compared to the CON, ACL, and TKA groups (Fig. 1C).

### Serum cytokine concentrations with (KL ≥ 3) and without (KL < 3) joint space narrowing

**Figure 2:**
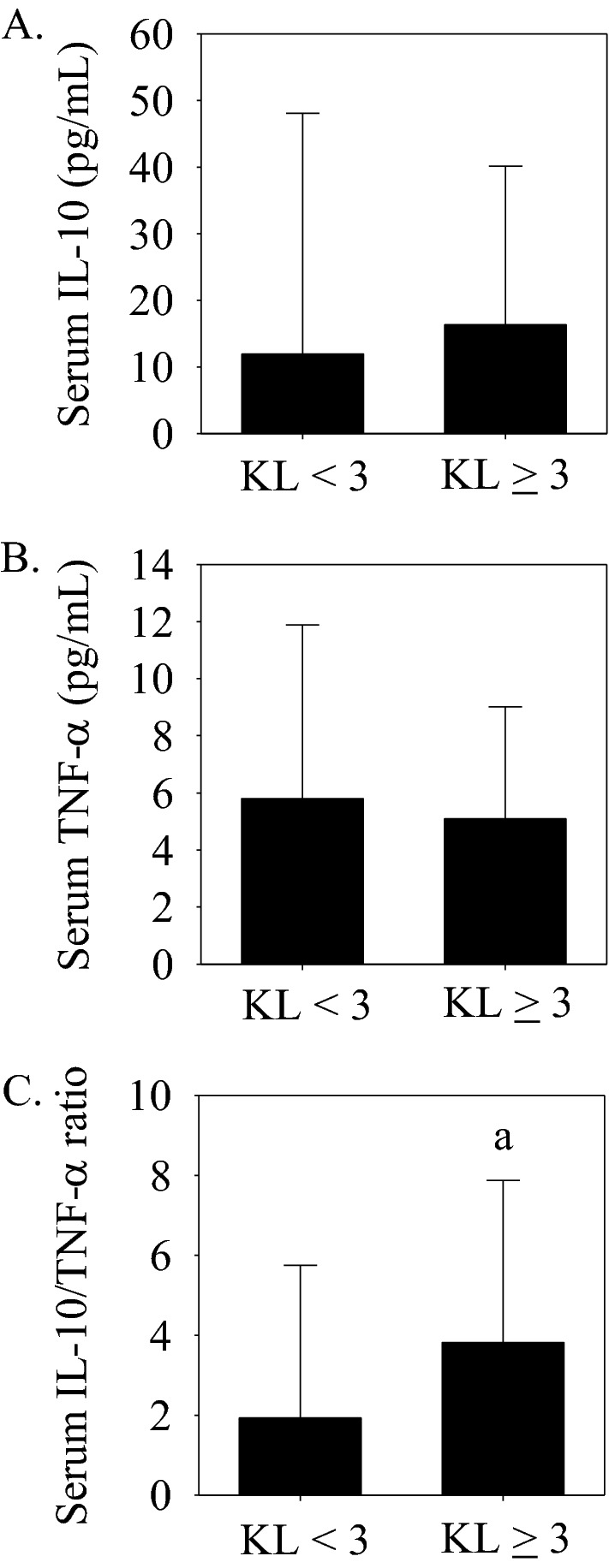
Serum IL-10 and TNF-α concentrations and the serum IL-10/TNF-α ratio without (KL grade < 3, *n* = 51) and with (KL grade ≥ 3, *n* = 79) radiographic evidence of knee joint space narrowing. (**A**) Serum IL-10 and (**B**) Serum TNF-α concentrations were not significantly different between groups. (**C**) The serum IL-10/TNF-α ratio was significantly (^a^*p* < 0.05 vs. KL grade < 3) higher in the KL grade ≥ 3 group. Data presented as median (interquartile range).

To extend those previously^26,37–39^, serum IL-10 and TNF-α concentrations and the serum IL-10/TNF-α ratio were compared between subjects with (i.e., KL grade ≥ 3 *n* = 79) and without (i.e., KL grade < 3, *n* = 51) radiographic evidence of joint space narrowing and independent from their initial classifications (i.e., CON, ACL, OA, or TKA). Serum IL-10 (Fig. 2A) and TNF-α (Fig. 2B) concentrations were not significantly different between KL grade groups (KL grade < 3 vs. ≥ 3), but the serum IL-10/TNF-α ratio was significantly higher in subjects with evidence of joint space narrowing (i.e., KL ≥ 3; Fig. 2C).

### Serum cytokine concentrations with moderate (KL = 3) compared to severe (KL = 4) knee joint space narrowing

**Figure 3:**
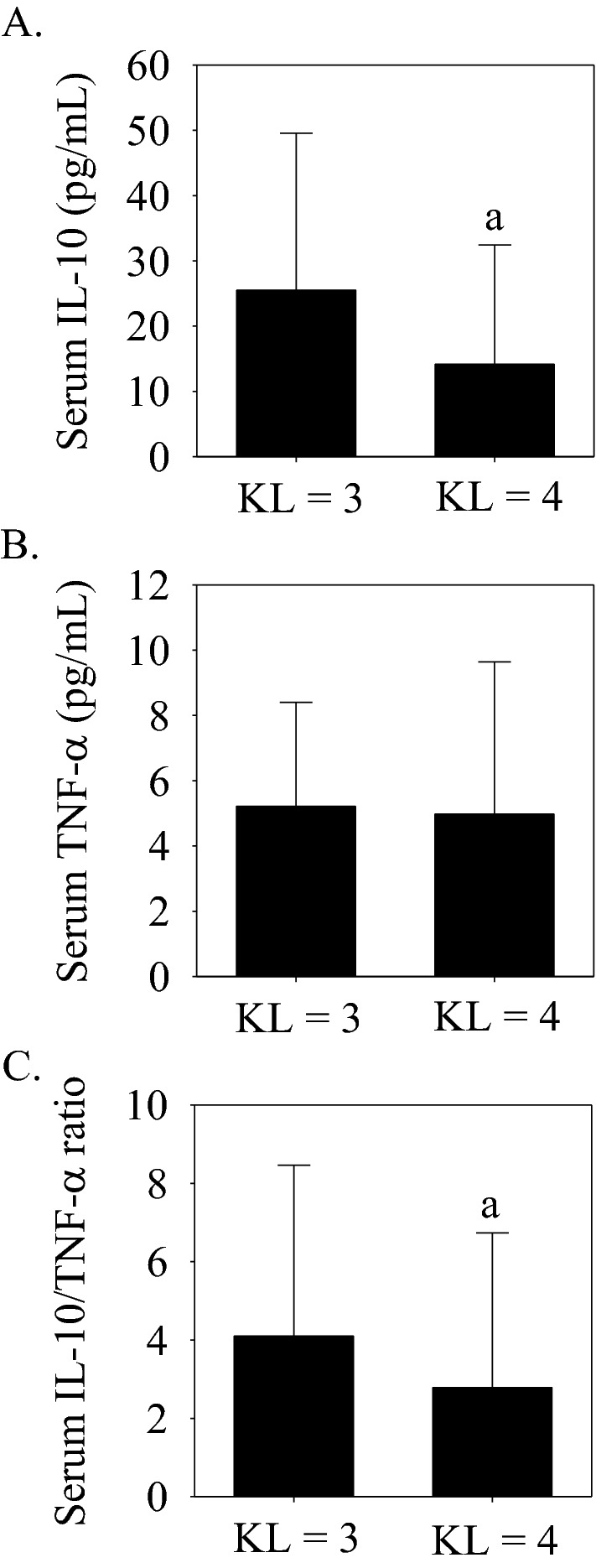
Serum IL-10 and TNF-α concentrations and the serum IL-10/TNF-α ratio with a KL grade = 3 (moderate knee osteoarthritis, ≤ 50% joint space narrowing, *n* = 49) compared to a KL grade = 4 (severe knee osteoarthritis, > 50% joint space narrowing, *n* = 30). (**A**) Serum IL-10 was significantly (^a^*p* < 0.05 vs. KL grade = 3) lower with a KL grade = 4. (**B**) Serum TNF-α concentrations were not significantly different between groups. (**C**) The serum IL-10/TNF-α ratio was significantly (^a^*p* < 0.05 vs. KL grade = 3) lower with a KL grade = 4. Data presented as median (interquartile range).

Another primary outcome of this study was the comparison of serum IL-10 and TNF-α concentrations and the IL-10/TNF-α ratio between moderate (i.e., KL = 3, ≤ 50% joint-space narrowing; *n* = 49) and severe (i.e., KL = 4, > 50% joint space narrowing; *n* = 30) knee joint space narrowing. Serum IL-10 concentrations and the serum IL-10/TNF-α ratio were lower and serum TNF-α was not significantly different with a KL grade of 4 compared to a grade 3 (Fig. 3A–C), implying a compromise in the circulating anti-inflammatory status with severe knee osteoarthritis.

### Correlations

As a group (*n* = 218), age, height, body mass and BMI correlated (Table 3). However, age, height, body mass and BMI were not significantly correlated with serum IL-10 or TNF-α. Thus, although there were discrepancies in subject characteristics (such as gender, age, and BMI) between the CON, ACL, OA, and TKA groups, serum cytokines did not correlate with subject characteristics. TNF-α correlated (*p* < 0.05) with IL-10 as a group (*n* = 218; Table 3) and in each group separately (Table 4).

**Table 3:** Group correlation coefficients.

	Age	Height	Mass	BMI	IL-10
Height	− 0.25^a^				
Mass	0.32^a^	0.43^a^			
BMI	0.51^a^	− 0.08	0.84^a^		
IL-10	0.08	0.10	0.10	0.04	
TNF-α	− 0.01	0.16	0.12	0.02	0.44^a^

^a^*p* < 0.05, *n* = 218; Mass, body mass.

**Table 4:** Cytokine correlation coefficients in each group.

	IL-10
CON (*n* = 92)	
TNF-α	0.47^a^
ACL (*n* = 42)	
TNF-α	0.49^a^
OA (*n* = 60)	
TNF-α	0.53^a^
TKA (*n* = 24)	
TNF-α	0.52^b^

^a^*p* < 0.05; ^b^*p* = 0.05.

## Discussion

In this investigation, we provide original evidence that the circulating concentration of an anti-inflammatory cytokine is compromised following an anterior cruciate ligament injury. Despite unperturbed individual concentrations, the circulating pro- to -anti-inflammatory cytokine ratio shifted toward the latter with radiographic evidence of joint space narrowing knee osteoarthritis. However, upon further examination and delineation in osteoarthritic phenotypes (i.e., moderate vs. severe), this report illustrates a lower anti-inflammatory cytokine and IL-10/TNF-α ratio in the absence of a robust pro-inflammatory cytokine deviation in the circulation with severe compared to moderate knee osteoarthritis.

Lower circulating IL-10 corresponds with higher joint effusion of TNF-α, localized and systemic indices of cartilage degeneration, and greater histological evidence of joint osteoarthritis following anterior cruciate ligament transection in experimental rabbits^40^. In humans, a lower serum IL-10 is arguably mirrored by a decrease in synovial fluid IL-10^12,41^, which in turn, could compromise joint integrity by exacerbating blood-induced cartilage damage^42^, and as demonstrated in vitro, minimize the protection against pro-inflammatory cytokine induced damage^22–25^. Therefore, in the absence of a robust TNF-α alteration, it is plausible that a lower serum IL-10 concentration following an anterior cruciate ligament injury could be detrimental to the joint structure by compromising the anti-inflammatory protection against pro-inflammatory cytokine-mediated cartilage loss and consequentially increase the risk of developing knee osteoarthritis. However, evidence supporting this premise remains elusive^11^, but if demonstrated, could provide evidence of a systemic biomarker intended to improve prognostic potential and the subsequent rationale necessary for a personalized approach of preventative medicine following an anterior cruciate ligament injury.

In the OA group, the unperturbed serum IL-10 corresponds with a lower serum TNF-α concentration compared to the CON group. Considering the anti-inflammatory property of IL-10 to down-regulate TNF-α production^43^, these results intuitively imply that a collected anti-inflammatory cytokine status associates with a lower pro-inflammatory cytokine concentration in the circulation with moderate knee osteoarthritis. However, the correlational results are not consistent with this assumption and imply an alternative explanation. Specifically, there is a positive correlation between TNF-α and IL-10 in the OA group, in each group separately, and in all subjects combined, suggesting an increase in IL-10 with increasing TNF-α. This finding, however, is not consistent with previous results as data provided by Koh and colleagues^15^ failed to demonstrate a correlation between circulating IL-10 and TNF-α in subjects with an anterior cruciate ligament injury, subjects with end-stage knee osteoarthritis, and in anterior cruciate ligament injuried and end-stage knee osteoarthritis subjects combined.

To explore the pro- to anti-inflammatory cytokine balance, we compared the IL-10/TNF-α ratio between groups. The IL-10/TNF-α ratio is considered as an anti-inflammatory index^29,44^ and is significantly elevated in the OA compared to the CON, ACL, and TKA groups. An elevation in the IL-10/TNF-α ratio (circulating and peripheral blood mononuclear cell messenger RNA) prevents or attenuates joint degeneration in experimental animals with post-traumatic osteoarthritis^40^ and associates with fewer post-operative complications in cardiac surgery patients^45^. However, an increase in the ratio is not consistently associated with improved outcomes as transient deviations or sustained elevations in the IL-10/TNF-α ratio could also represent hyper-inflammation or immunosuppression and relate to poor outcomes in pathophysiological conditions, such as sepsis^29^. The disparate results from the aforementioned studies underscore the importance of a cautious interpretation when examining the IL-10/TNF-α ratio, while herein illustrate an augmentation in circulating IL-10 for a given TNF-α concentration in the OA group predominately characterized by moderate knee osteoarthritis (i.e., KL grade = 3).

Research endeavors and data continue to mount pertaining to the regulatory role of cytokines on osteoarthritis development and progression^15,19,21,26,27,33,38,46–49^. Here, we performed two separate analyses to further our understanding of the association between the pro- to anti-inflammatory cytokine balance and the severity in knee osteoarthritis. In the first analysis, serum IL-10 and TNF-α concentrations and the serum IL-10/TNF-α ratio are compared between those with (i.e., KL ≥ 3, *n* = 79) to those without (KL < 3, *n* = 51) radiographic evidence of knee joint space narrowing and independent from initial groupings (i.e., CON, ACL, OA, and TKA). In those with available radiographs (*n* = 130), the serum IL-10/TNF-α ratio is significantly elevated with gross joint space narrowing (i.e., KL ≥ 3) while serum IL-10 and TNF-α concentrations are not significantly different with compared to without joint space narrowing. This finding implies that the systemic pro- to anti-inflammatory cytokine balance might be more sensitive to gross alterations in joint space narrowing and disease severity than individual TNF-α and IL-10 concentrations.

In the second analysis, we limited the comparison to osteoarthritic subtypes with contrasting evidence in disease severity or joint space narrowing, and again, independent from initial groupings. Specifically, serum IL-10 and TNF-α concentrations and the serum IL-10/TNF-α ratio are compared between moderate (KL = 3, ≤ 50% joint space narrowing) and severe (KL = 4, > 50% joint space narrowing) knee osteoarthritis. Results from this analysis indicate that serum IL-10 concentrations and the serum IL-10/TNF-α ratio are lower with severe knee osteoarthritis while the serum TNF-α concentration is not significantly different between disease severity subgroups. These findings collectively suggest an elevation in the serum IL-10/TNF-α ratio with gross joint space narrowing, but a lower serum IL-10 concentration and IL-10/TNF-α ratio with severe compared to moderate knee osteoarthritis. Considering cartilage loss occurs before the radiographic manifestation of joint space narrowing^50,51^, future research associating the IL-10/TNF-α ratio with cartilage loss and radiographic evidence of joint space narrowing could reveal a systemic biomarker sensitive to disease development and progression that aids in the clinical decision process. Also, although identification of the potential source(s) responsible for modulating serum IL-10 is beyond the scope of the present investigation, it is plausible that a lower anti-inflammatory cytokine concentration with severe or end-stage knee osteoarthritis—when the majority of the cartilage damage has already occurred—associates with an alteration in the number of infiltrating mononuclear cells (e.g., CD4 + and CD68 + cells)^28,52–56^, chondrocytes^27,57^, and other sources that contribute to its production.

In addition to those discussed above, there are other study limitations worthy of discussion. First, more sensitive imaging tools could improve the accuracy in linking disease severity or joint trauma with circulating cytokines, although this could be questionable^11^. Along these lines, magnetic resonance imaging was not utilized in the ACL group to evaluate additional trauma to the knee joint, but rather, was assessed intraoperatively during arthroscopy. Therefore, it is plausible that additional trauma to knee joint was present in the ACL group that was not captured during surgery. Second, stringent inclusion and exclusion criteria are implemented to minimize the impact of comorbidities on study outcomes, but in turn, could hinder the generalizability of the results to a broader population. Third, we did not delineate primary osteoarthritis from secondary causes of osteoarthritis (e.g., post-traumatic injury) in the OA and TKA groups. Fourth, although subject characteristics were not correlated with circulating cytokines or cytokine ratios, it is plausible that various subject characterstics could be confounding the results. Next, this study did not concurrently examine circulating and localized cytokine concentrations. Finally, although subject characteristics were not significantly different between the osteoarthritis groups and subgroups, including a non-osteoarthritis group with similar demographics to the osteoarthritic groups could reveal a difference in circulating cytokine concentrations with general knee osteoarthritis^58^.

## Conclusion

This investigation provides unique data illustrating an increase in the serum IL-10/TNF-α ratio with robust joint space narrowing. Differentiating moderate from severe knee osteoarthritis, however, revealed a lower serum IL-10 concentration and serum IL-10/TNF-α ratio with severe knee osteoarthritis. This report also provides evidence that serum IL-10 concentrations are lower while serum TNF-α concentrations and the serum IL-10/TNF-α ratio are unperturbed in subjects predisposed to developing knee osteoarthritis following ligamentous trauma. Based on these findings, we conclude that serum IL-10 concentrations are compromised without significant serum TNF-α deviations in subjects predisposed to developing knee osteoarthritis following ligamentous trauma and in subjects with severe radiographic evidence of knee joint space narrowing. Future research assessing the clinical utility of the serum IL-10/TNF-α ratio and other cytokine and cytokine ratios as systemic biomarkers capable of phenotypically differentiating the severity in knee osteoarthritis is warranted.

## Data Availability

The datasets generated and analyzed during this study are not publicly available due data containing potentially identifying or sensitive patient information but are available from the corresponding author upon reasonable request.

## Abbreviations

ACL: Anterior cruciate ligament
BMI: Body mass index
CON: Control
IL-10: Interleukin-10
KL: Kellgren–Lawrence
OA: Osteoarthritis
TKA: Total knee arthroplasty
TNF-α: Tumor necrosis factor-α
